# Shiga Toxin–Producing *Escherichia coli–*Associated Hemolytic Uremic Syndrome in Adult Kidney Transplant Recipients

**DOI:** 10.1016/j.ekir.2025.08.004

**Published:** 2025-08-11

**Authors:** Manal Mazloum, Pierre Trémolières, Nesrine Baili, Cédric Rafat, Hannah Kaminski, Nassim Kamar, Edouard Lefevre, Dominique Bertrand, Charlene Levi, Sophie Chauvet, François Provôt, Roxane Gamot, Pierre-François Westeel, Sophie Caillard, Nicolas Bouvier, Fatouma Touré, Simon Ville, Moglie Le Quintrec, Dany Anglicheau, Rebecca Sberro-Soussan, Frank Martinez, Christophe Legendre, Julien Zuber, Stéphane Bonacorsi, Patricia Mariani, Aurélie Cointe, Anne Scemla

**Affiliations:** 1 Department of Kidney and Metabolic Diseases, Transplantation and Clinical Immunology, Necker Hospital, Assistance Publique-Hôpitaux de Paris, Paris, France; 2 Department of Nephrology, Dialysis and Transplantation, Lapeyronie Center University Hospital, Montpellier, France; 3 Department of Nephrology and Transplantation, CHRU de Nancy - Hôpitaux de Brabois, Vandœuvre-lès-Nancy, France; 4 Department of Nephrology, Tenon Hospital, Paris, France; 5 Department of Nephrology, Transplantation, Dialysis and Apheresis, Pellegrin University Hospital, Bordeaux, France; 6 Department of Nephrology and Organ Transplantation – Axe TImE, Toulouse Rangueil University Hospital, INSERM UMR 1291, Toulouse Institute for Infectious and Inflammatory Diseases (Infinity), University Paul Sabatier, Toulouse, France; 7 Department of Nephrology, Kremlin Bicêtre University Hospital, Le Kremlin-Bicêtre, France; 8 Department of Nephrology and Transplantation, Hospital Center University De Rouen, Rouen, France; 9 Department of Transplantation, Nephrology, and Clinical Immunology, Edouard Herriot Hospital, Lyon, France; 10 Department of Nephrology, European Hospital Georges Pompidou, Assistance Publique-Hôpitaux de Paris, Paris, France; 11 Department of Nephrology, Hospital Center University De Lille, Lille, France; 12 Department of Nephrology, CHRU Hospitals of Tours, Tours, France; 13 Department of Nephrology, CHU Amiens Picardie Hélisurface, Salouël, France; 14 Department of Nephrology and Transplantation, CHU Strasbourg, Strasbourg, France; 15 Department of Nephrology, Transplantation, Dialysis, University of Caen Normandie Hospital Center, Caen, France; 16 Department of Nephrology, Dialysis and Transplantation, CHU Dupuytren, Limoges, France; 17 Department of Adult Nephrology and Immunology, Centre Hospitalier Universitaire de Nantes, Nantes, France; 18 Department of Microbiology, Reference National Center of Escherichia coli, Hospital Robert Debré AP-HP, Paris, France; 19 Université Paris Cité, INSERM, IAME, Paris, France

**Keywords:** hemolytic uremic syndrome, kidney transplantation, Shiga-toxin *Escherichia coli*, thrombotic microangiopathy

## Abstract

**Introduction:**

Shiga toxin (Stx)-producing *Escherichia coli (E coli)-*associated hemolytic uremic syndrome (STEC-HUS) is an acquired form of thrombotic microangiopathy (TMA) caused by food and waterborne infection. It remains the leading cause of acute renal injury (AKI) in children. In adults, STEC-HUS is less common but often more severe. Data on STEC-HUS in kidney transplant recipients (KTRs) are limited.

**Methods:**

We conducted a retrospective study involving adult KTRs diagnosed with STEC-HUS between January 2012 and April 2022 across 15 French nephrology centers. We aimed to characterize the clinical, biological, and microbiological features of STEC-HUS in this population, its management, and outcomes.

**Results:**

A total of 35 adult KTRs were included. The annual incidence was estimated at 14.4 cases per 100,000 KTRs. Six and 29 patients presented with localized intrarenal or systemic TMA, respectively. The median time from transplantation to STEC-HUS diagnosis was 3 (1.2–6.2) years and the mean age at diagnosis was 57 ± 13 years. Neurological and cardiac complications occurred exclusively in patients with systemic TMA. AKI and significant proteinuria were observed in 89% and 94% of patients, respectively. All patients requiring dialysis exhibited systemic TMA (38%). Treatment included azithromycin, eculizumab, and conversion from calcineurin inhibitors (CNIs) to belatacept. Graft loss and mortality rates were 26% and 9%, respectively.

**Conclusion:**

This study highlights that STEC-HUS represents a serious complication in KTRs, significantly impairing renal and overall survival. It should be systematically considered in cases of *de novo* posttransplant TMA, whether in the absence of prodromal diarrhea or in localized intra-renal presentations.

## Graphical abstract

**Figure:**
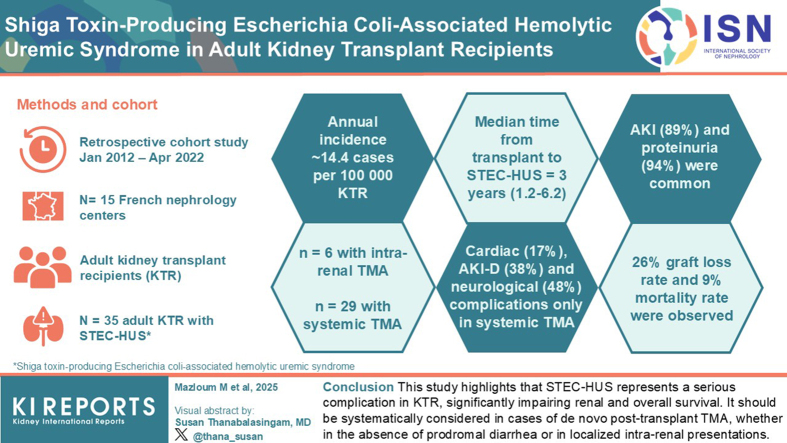

TMA refers to a clinico-biological syndrome characterized by the association of thrombocytopenia, microangiopathic hemolytic anemia and acute ischemic organ damage, acute kidney injury (AKI) being the most frequent manifestation.^1^ In the general population, it remains a rare but potentially life-threatening condition, resulting from acute and diffuse microvascular thrombotic occlusion.^1^ In the field of kidney transplantation, TMA affects 1% to 14% of KTRs,^2^ resulting in long-term graft loss rate of 30% to 40%.^3^ Posttransplant TMA may occur either as a *de novo* or recurrent disease, as usually classified.^2^ Although atypical HUS (aHUS) represents the first cause of recurrent posttransplant TMA, etiologies of *de novo* posttransplant TMA commonly include immunosuppressive medications (i.e., CNI and mTOR inhibitors), antibody-mediated rejection, viral infections, and malignant hypertension.^2^^,^^4^, ^5^, ^6^

STEC-HUS, formerly referred to as typical HUS, is a distinct clinical entity of TMA.^7^ As opposed to aHUS which results from genetic or acquired dysregulation of the alternative complement pathway, STEC are foodborne and waterborne pathogens mainly originating from fecal excretion of cattle and other ruminants. The global prevalence of STEC-HUS has been estimated at about 4000 annual cases.^8^ Although STEC-HUS is more frequent in the pediatric population and still represents the leading cause of AKI in children aged < 3 years,^9^ clinical presentation is more severe in adults^10^^,^^11^ and the related risk of death has been found to be significantly increased after the age of 60 years.^12^ Clinical presentation typically starts with bloody diarrhea between 4 to 7 days after contamination. AKI usually occurs within a period of 5 to 10 days after the onset of symptoms, requiring renal replacement therapy (RRT) in nearly 40% of patients, and about 70% of cases fully recover.^13^^,^^14^ Patients with STEC-HUS may present with multiple organ failure,^14^ leading to death in about 2% and 20% of pediatric and adults cases, respectively.^13^^,^^14^ Diagnosis of STEC-HUS is confirmed in the presence of biological TMA associated with positive stool culture for STEC and/or positive stool polymerase chain reaction (PCR) for gene encoding Stx.^9^ Despite significant progress in the understanding of STEC-HUS pathophysiology mainly based on the endothelial toxicity of Stx, supportive therapy remains the current mainstay, including fluid management, RRT, and treatment of damaged organs.^15^

If characteristics of STEC-HUS have been well-documented in the general population, data regarding this disorder in the population of organ transplant recipients, more specifically of KTRs, is remarkably scarce. To date, 8 cases of STEC-HUS have been reported in solid organ^16^, ^17^, ^18^ and bone marrow^19^ transplant recipients, of which only 2 cases were in KTRs from France^16^ and Colombia,^17^ with different presentations, management, and outcomes. Both cases presented with watery and nonbloody diarrhea, AKI, and typical biological signs of TMA, leading to graft loss in 1 case^16^ and death in the other case.^17^ Interestingly, in a recent cohort study of 96 adult cases of STEC-HUS from the French Reference Center for Thrombotic Microangiopathies, immunodeficiency, which concerned about one-third of patients, was found as a major risk factor for death.^11^ Of note, 8 patients with a history of bone marrow or solid organ transplant were included, 5 of whom were KTRs.^11^ Therefore, these data combined with the known increased susceptibility of immunocompromised patients to fatal systemic infections and the high affinity of Stx for the renal endothelium may encourage nephrologists to better characterize this disorder in KTRs. Therefore, we conducted a French retrospective study aiming to determine the specific features of STEC-HUS in adult KTRs. We first described the clinical, biological, and histological presentation, as well as the microbiological relative distribution in this population. We also reported the different therapeutic approaches and prognosis regarding kidney graft and patient survival.

## Methods

### Study Population

We conducted a retrospective and multicenter observational study reporting STEC-HUS in adult KTRs (aged ≥ 18 years at the time of diagnosis) occurring between January 2012 and April 2022 from 15 French nephrology centers. For all patients, the diagnosis of STEC-HUS was based on the association of the following 3 criteria: (i) STEC infection confirmed by a positive stool multiplex PCR test for STEC; (ii) biological evidence of renal involvement, that is, AKI, as defined below, and/or proteinuria ≥ 0.5 g/g; and (iii) and systemic and/or localized TMA, as defined next.

AKI was defined and graded according to Kidney Disease Improving Global Outcomes criteria (2012).^20^ Systemic TMA was defined as the presence of thrombocytopenia (platelet levels < 150,000 cells/μl) and/or microangiopathic hemolytic anemia (presence of schistocytes on blood smear and/or elevated lactate dehydrogenase serum levels and/or decreased or undetectable serum haptoglobin). Histopathological features of TMA included thrombotic lesions and/or patterns of endothelial cell activation (mesangiolysis, endothelial swelling, microaneurysms, double contours of capillary walls, capillary wall thickening) and were classified according to glomerular and/or arteriolar thrombi location. Mixed TMA referred to the association of glomerular and arteriolar lesions of TMA. Patients who had a combined transplantation or a STEC infection without the 2 other criteria were excluded.

The included patients were then divided into 2 distinct groups. The first group named as “systemic TMA” included patients with systemic TMA and the second group named as “localized TMA” included patients with histological signs of TMA as previously described, with no biological features of systemic TMA. Thus, all patients with localized TMA were diagnosed after a for-cause kidney graft biopsy, initially indicated for AKI and/or proteinuria ≥ 0.5 g/g.

### Microbiological Data

Most patients’ stools with a multiplex PCR positive for STEC in local laboratories were analyzed in the National Reference Center for *E coli* at the Department of Microbiology of Robert Debré University Hospital, Paris, France. The 2 major types of Stx were detected using *stx1*- and *stx2*-specific PCR on stool as previously described.^21^ When positive, stool cultures were performed for isolation and characterization of the STEC strains.^21^ *E coli* strain was classified as undetermined if it could not be identified in culture.

### Data Collection

For each patient, data were collected from the available medical charts. The different centers were asked by e-mail to collect general information about the recipient (male or female sex, initial nephropathy, and medical history) and the transplantation (age, rank, dialysis before transplantation, donor source and age, and type of induction). The annual incidence rate was calculated as the number of included cases of STEC-HUS divided by the total number of KTRs followed-up with in the 15 participating centers between 2012 and 2022, which was provided by the medical and scientific activity report of the Agency of Biomedicine in 2023 (https://rapport-annuel.agence-biomedecine.fr).

Data also included general information about patients before diagnosis (baseline creatininemia and urine protein-to-creatinine ratio (UPCR), and history of acute rejection) and at diagnosis of STEC-HUS (age and hospitalization). Clinical manifestations reflecting the main organs involvement (renal, gastrointestinal, neurological, cardiac, and fever), biological results (hemoglobin level, platelet and white blood cell count, C-reactive protein, schistocytes, lactate dehydrogenase, haptoglobin, creatininemia, and proteinuria), specific complement analysis and microbiological characteristics (*E coli* serogroups and Stx types) at diagnosis of STEC-HUS were collected. The presence of features of histological TMA (glomerular and or/arteriolar TMA) was indicated when a kidney graft biopsy was performed. Data concerning the therapeutic management and the patients’ and graft outcome (graft loss, acute rejection, and transplantectomy) were extracted.

### Statistical Analyses

Data were analyzed using GraphPad Prism V10 Software (GraphPad Prism, La Jolla, CA). Categorical variables were expressed as number (percentages) and Fisher exact test was used for comparisons. Continuous variables were expressed as mean ± SD when normally distributed and as median (interquartile range: 25th–75th percentile) in case of skewed distribution. We used unpaired *t* test and nonparametric Mann-Whitney test for comparisons. Values of *P* < 0.05 were considered statistically significant.

### Ethics

This study was approved by the French Ethic Committee of Research in Infectious and Tropical Diseases (N° CER-MIT : 2024-0303-2), in accordance with the French data protection authority (CNIL registration number 2232874).

## Results

### Baseline Characteristics

**Figure 1:**
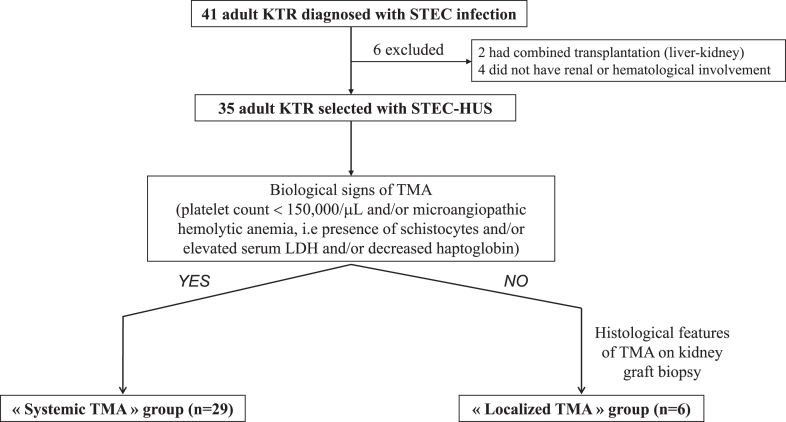
Flow chart. HUS, hemolytic uremic syndrome; KTRs, kidney transplant recipients; STEC, Shiga toxin-producing *Escherichia coli*; TMA, thrombotic microangiopathy.

A total of 35 KTRs diagnosed with STEC-HUS between 2012 and 2022 were included in the final analysis. The flow chart is presented in Figure 1. Based on the total number of KTRs followed-up with during this period provided by the Agency of Biomedicine, we estimated the annual incidence rate of STEC-HUS to be 14.4 per 100,000 KTRs. Among the included KTRs, 6 and 29 patients were classified as “localized TMA” and “systemic TMA”, respectively. Their initial characteristics are presented in Table 1. At the time of kidney transplantation, these characteristics were similar between the 2 groups. Overall, 17 were female (49%) and glomerulonephritis was the most frequent native nephropathy (*n* = 7; 36%), whereas no case of native HUS was found. Regarding renal function, the median values of baseline serum creatinine level and UPCR, measured from 3 to 6 months before STEC-HUS diagnosis, were 1.5 mg/dl and 0.2 g/g, respectively. Median baseline UPCR was significantly higher in the “localized TMA” group (0.8 [0.5–2.1] g/g vs. 0.1 [0–0.06] g/g; *P* = 0.01).

**Table 1:** Baseline characteristics before diagnosis of STEC-HUS

Characteristics	Localized TMA (*n* = 6)	Systemic TMA (*n* = 29)	All patients (*N* = 35)	*P*-value^a^
Male sex	4/6 (67%)	14/29 (48%)	18/35 (51%)	0.66
Native nephropathy				
Cystic kidney disease	2/6 (33%)	4/29 (14%)	6/35 (17%)	0.27
Diabetic nephropathy	1/6 (17%)	5/29 (17%)	6/35 (17%)	0.99
Vascular nephropathy	0/6 (0%)	1/29 (3%)	1/35 (3%)	0.99
Glomerulonephritis	2/6 (33%)	5/29 (17%)	7/35 (36%)	0.58
Genetic glomerular disease	0/6 (0%)	4/29 (14%)	4/35 (11%)	0.99
CAKUT or uropathy	0/6 (0%)	3/29 (10%)	3/35 (9%)	0.99
HUS	0/6 (0%)	0/29 (0%)	0/35 (%)	0.99
Other	1/6 (17%)	1/29 (3%)	2/35 (6%)	0.32
Undetermined	0/6 (0%)	6/29 (21%)	6/35 (17%)	0.56
Preemptive transplantation	1/6 (17%)	8/29 (28%)	9/35 (26%)	0.99
ABO incompatible transplantation	0/6 (0%)	2/29 (7%)	2/35 (6%)	0.99
First kidney transplantation	6/6 (100%)	25/29 (86%)	31/35 (89%)	0.99
Living donor	3/6 (50%)	7/29 (24%)	10/35 (29%)	0.32
Donor age (yr)	54 ± 21	56 ± 14	56 ± 15	0.75
Induction immunosuppressive therapy				
Basiliximab	3/6 (50%)	10/26 (38%)	13/32 (41%)	0.67
Antithymocyte globulins	3/6 (50%)	16/26 (62%)	19/32 (59%)	0.67
Intravenous immunoglobulins	2/6 (33%)	3/29 (10%)	5/35 (14%)	0.20
Rituximab	0/6 (0%)	3/29 (10%)	3/35 (9%)	0.99
Plasma exchange	0/6 (0%)	3/29 (10%)	3/35 (9%)	0.99
Diabetes mellitus	2/6 (33%)	10/29 (34%)	12/35 (34%)	0.99
Hypertension	5/6 (83%)	24/27 (89%)	29/33 (88%)	0.99
Cancer	2/6 (33%)	6/29 (21%)	8/35 (23%)	0.60
Major cardiovascular events^b^	3/6 (50%)	6/26 (23%)	9/32 (28%)	0.31
History of acute rejection	2/6 (33%)	2/29 (7%)	4/35 (11%)	0.13
Serum creatinine level (mg/dl)	1.9 (1.6–2.3)	1.5 (1.2–1.9)	1.5 (1.4–2.0)	0.06
UPCR (g/g)	0.8 (0.5–2.1)	0.1 (0–0.6)	0.2 (0.1–0.6)	0.01

CAKUT, congenital anomalies of the kidney and urinary tract; HUS, hemolytic uremic syndrome; STEC, Shiga toxin-producing *Escherichia coli*; TMA, thrombotic microangiopathy; UPCR, urine protein-to-creatinine ratio.

Categorical variables are described as numbers (%) and continuous variables are described as mean ± SD or median (interquartile range), as appropriate.

a *P*-value represents tests of significance from *t* test, Mann-Whitney test, or Fisher exact test, as appropriate.

b Major cardiovascular events include ischemic cardiomyopathy and stroke.

### Clinical Presentation and Biological Features at Diagnosis of STEC-HUS

Clinical data at the time of diagnosis of STEC-HUS are shown in Supplementary Table S1. Patients were diagnosed with STEC-HUS at a mean age of 57 ± 13 years, in a median time of 3 (range: 1.2–6.2) years since kidney transplantation. The median time between symptom onset and diagnosis of STEC-HUS was 11 (7–23) days, with a nonsignificant trend toward delayed diagnosis in patients with localized TMA (15 days, range: 10–25) as compared with patients with systemic TMA (10 days, range: 6–26), possibly because of a milder clinical presentation. Regarding maintenance immunosuppression, more patients were receiving belatacept at diagnosis of STEC-HUS (2/6; 33%) in the “localized TMA” group, without significant difference with the “systemic TMA” group (1/29; 3%).

Extrarenal manifestations in both groups are detailed in Supplementary Table S2. In total, 4 of 35 patients (11%) did not present with diarrhea. Most patients experienced nonbloody diarrhea at initial presentation (24/35; 69%). Neurological and cardiac manifestations and fever were only found in patients with systemic TMA, in 14 of 29 (48%), 5 of 29 (17%), and 7 and 29 (24%), respectively.

Characteristics of renal presentation are specifically compared in both groups in Table 2. At diagnosis of STEC-HUS, renal presentation was substantially more severe in the systemic TMA group, including oligoanuria (8/29; 28%), hematuria (9/19; 47%) and malignant hypertension (7/29; 24%). In total, 31 of 35 of patients (89%) initially had AKI, with a median serum creatinine level of 2.7 (2.2–4.2) mg/dl: using the KDIGO criteria, most patients were classified at stage 1 in localized TMA group (4/6 ; 67%) and at stage 3 in systemic TMA group (12/29; 41%). Nearly all patients presented with significant proteinuria (33/35; 94%) and the median values of UPCR at diagnosis were similar between the 2 groups (*P* = 0.20). In total, a kidney graft biopsy was performed in 23 of 35 (66%) patients at initial presentation: every biopsy revealed histological signs of TMA, mostly exclusive glomerular (13/35; 57%) or mixed TMA (9/23; 39%). As expected, hematological parameters detailed in Supplementary Table S3 were significantly altered in the systemic TMA group, in which the median platelet count was 97 (71–137) × 10^9^ cells and schistocytes were found in 18 of 29 patients (62%).

**Table 2:** Clinical, biological and histological features of renal presentation at diagnosis of STEC-HUS

Characteristics	Localized TMA (*n* = 6)	Systemic TMA (*n* = 29)	All patients (*N* = 35)	*P*-value^a^
Oligo-anuria	0/6 (0%)	8/29 (28%)	8/35 (23%)	0.30
Hematuria	0/2 (0%)	9/19 (47%)	9/21 (43%)	0.49
Malignant hypertension	0/6 (0%)	7/29 (24%)	7/35 (20%)	0.31
Serum creatinine level (mg/dl)	2.5 (2.0–2.8)	2.8 (2.3–4.8)	2.7 (2.2–4.2)	0.35
Acute kidney injury	4/6 (67%)	27/29 (93%)	31/35 (89%)	0.13
KDIGO classification				
Stage 1	4/6 (67%)	11/29 (38%)	15/35 (40%)	0.20
Stage 2	0/6 (0%)	4/29 (14%)	4/35 (11%)	0.99
Stage 3	0/6 (0%)	12/29 (41%)	12/35 (34%)	0.07
Proteinuria (i.e., ⩾ 0.5 g/g)	6/6 (100%)	27/29 (93%)	33/35 (94%)	0.99
UPCR (g/g)	1.4 (1.1–2.8)	2.4 (1.3–5.2)	2.3 (1.3–4.7)	0.20
Kidney graft biopsy	6/6 (100%)	17/29 (59%)	23/35 (66%)	0.07
Exclusive glomerular TMA	3/6 (50%)	10/17 (59%)	13/23 (57%)	0.99
Exclusive arteriolar TMA	0/6 (0%)	1/17 (6%)	1/23 (4%)	0.99
Mixed TMA	3/6 (50%)	6/17 (35%)	9/23 (39%)	0.64

HUS, hemolytic uremic syndrome; KDIGO, Kidney Disease: Improving Global Outcomes; STEC, Shiga toxin-producing *Escherichia coli*; TMA, thrombotic microangiopathy; UPCR, urine protein-to-creatinine ratio.

Categorical variables are described as numbers (%) and continuous variables are described as median (interquartile range).

a *P*-value represents tests of significance from Mann-Whitney test, or Fisher exact test, as appropriate.

### Complement Analysis

Complement analysis was performed in some patients at the diagnosis of STEC-HUS. Median plasma levels of C3 and C4 were measured at 0.85 (0.71–1.0) g/l and 0.25 (0.19–0.29) g/l, respectively. Only 1 of 28 patients had low plasma C3 levels. CD46 expression on leucocytes, plasma dosages of complement factor H and complement factor I measured in 8 of 35 patients were normal. No anti–complement factor H autoantibodies were detected in the 17 tested patients. Screening for complement gene mutations (complement factor H and I, CD46, complement factor B, and thrombomodulin) was performed in 13 of 35 patients and found a heterozygous mutation in the complement factor H gene in 1 patient.

### Microbiological Data

**Figure 2:**
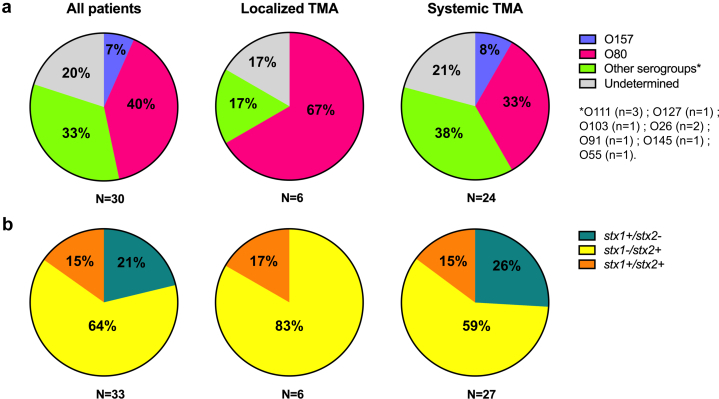
Distribution of (a) *E coli* serogroups and (b) Shigatoxin genotypes. TMA, thrombotic microangiopathy.

We then analyzed the distribution of *E coli* serogroups and Stx types (Figure 2). Data of *E coli* serogroups were missing in 5 of 35 patients (14%) and serogroups were undetermined in 6 of 30 patients (20%). The *E coli* serogroup O80 was the most prevalent, affecting 12 of 30 patients (40%), followed by the serogroup O111 (3/30 [10%]), O157 (2/30 [7%]), O26 (2/30 [7%]), and O127, O103, O91, O145, O55 (1/30, [3%] each). In the “localized TMA” group, 4 (67%) of patients were infected with O80 strain whereas O157 strain was not found. Among the 33 patients in which Stx type was determined, *stx1-/stx2+* was the most common profile (21/33; 64%), affecting 83% (5/6) and 59% (16/27) patients in localized and systemic TMA groups, respectively. Of note, *stx1* alone was detected in 7 of 27 patients (26%) with systemic TMA. The distribution of *E coli* strains and Stx types was not statistically different between the 2 groups (data not shown).

### Treatment and Outcomes

The management of STEC-HUS is presented in detail for both groups in Table 3. All patients presenting with systemic TMA were hospitalized as compared with 2/6 (33%) patients with localized TMA, for a median duration of 23 (15–39) days and 6 (2–10) days, respectively (*P* = 0.04). Immunosuppressive regimen was changed in 23 of 35 (66%) patients, mainly consisting of CNI discontinuation in 19 of 35 patients (54%), to limit the extension of TMA process. Among them, 12/35 (34%) patients were converted to belatacept. Tacrolimus was converted to ciclosporin in 2 patients and mycophenolate mofetil was converted to azathioprine in 1 patient for profuse diarrhea related to STEC infection.

**Table 3:** Management at diagnosis of STEC-HUS

Characteristics	Localized TMA (*n* = 6)	Systemic TMA (*n* = 29)	All patients (*N* = 35)	*P*-value^a^
Hospitalization	2/6 (33%)	29/29 (100%)	31/25 (89%)	0.003
Length of hospital stay (d)	6 (2–10)	23 (15–39)	22 (14–38)	0.04
Change in immunosuppression	4/6 (67%)	19/29 (70%)	23/35 (66%)	0.99
CNI withdrawal	4/6 (67%)	15/29 (52%)	19/35 (54%)	0.67
Switch CNI-belatacept	4/6 (67%)	8/29 (28%)	12/35 (34%)	0.15
Antibiotics	4/6 (67%)	20/29 (69%)	24/35 (69%)	0.99
Azithromycin	4/6 (67%)	12/29 (41%)	16/35 (46%)	0.38
Other antibiotics	0/6 (0%)	8/29 (28%)	8/35 (23%)	
Eculizumab	2/6 (33%)	12/29 (41%)	14/35 (40%)	0.99
Plasmapheresis	0/6 (0%)	11/29 (38%)	11/35 (31%)	0.15
Renal replacement therapy	0/6 (0%)	11/29 (38%)	11/35 (31%)	0.15
Red blood cell transfusion	1/6 (17%)	14/29 (48%)	15/35 (43%)	0.21
Platelet transfusion	0/6 (0%)	1/29 (3%)	1/35 (3%)	0.99

CNI, calcineurin inhibitor; HUS, hemolytic uremic syndrome; STEC, Shiga toxin-producing *Escherichia coli*; TMA, thrombotic microangiopathy.

Categorical variables are described as numbers (%) and continuous variables are described as median (interquartile range), as appropriate.

a *P*-value represents tests of significance from Mann-Whitney test or Fisher exact test, as appropriate.

In total, 24 of 35 patients (69%) were initially treated with antibiotics, including 16 of 35 (46%) with azithromycin. Besides, 7 patients received β-lactam drugs, and 1 patient received metronidazole for concomitant *Giardia* infection. In the “systemic TMA” group specifically, 11 of 29 patients (38%) received plasma exchanges, 7 of whom had initial neurological manifestations. Moreover, eculizumab was administered to 14 of 35 patients (40%) with a similar frequency in both groups and was associated with plasma exchanges in 6 patients among patients with systemic TMA. RRT was initiated in 11 of 29 patients (38%) presenting with systemic TMA, whereas no patient with localized TMA required RRT. In total, 16 of 35 patients (46%) received transfusion support.

At 3 months, among patients with a persistent functional graft, the median serum creatinine level was 2.0 (1.4–2.4) mg/dl and UPCR was 0.9 (0.3–2.0) g/g, without statistical difference between the 2 groups. The mean difference in serum creatinine level between 3 months after diagnosis of STEC-HUS and baseline was 0.2 ± 0.4 and 1.1 ± 1.7 within the “localized” and “systemic TMA” group, respectively (*P* = 0.24) (Supplementary Figure S1A).

At last follow-up, among patients with a persistent functional graft, the median serum creatinine level was significantly higher in the “localized TMA” group (2.1 mg/dl, range: 1.9–3.1 vs. 1.6 mg/dl, range: 1.2–1.9; *P* = 0.04). The mean difference in serum creatinine level between last follow-up and baseline was 0.5 ± 0.3 and 0.3 ± 0.8 within “localized” and “systemic TMA” group, respectively (*P* = 0.66) (Supplementary Figure S1B). These findings were probably explained by the exclusion of the 9 patients experiencing graft loss in the “systemic TMA” group.

Renal and global outcome data are detailed in Table 4. After a median follow-up of 22 (6–48) months, death occurred in 3 of 35 patients (9%), all belonging to the “systemic TMA” group and in a median time of 53 (6–270) days since diagnosis of STEC-HUS. Among them, all patients had initial neurological signs, and 2 patients were dialysis-dependent. Early death occurred of 1 patient 6 days after diagnosis of STEC-HUS, 1 patient died of cryptococcal infection, and 1 died of pneumonia and cytomegalovirus disease. In total, 9 of 35 patients (26%) experienced graft loss in a median time of 19 (1–55) days since diagnosis of STEC-HUS, all presented with systemic TMA. Among them, 7 required dialysis at initial presentation. Transplantectomy was performed for refractory TMA in 3 of 35 patients (9%) in a median time of 31 (28–33) days since diagnosis, and 1 patient had developed acute rejection after recovery from STEC-HUS.

**Table 4:** Outcome data

Characteristics	Localized TMA (*n* = 6)	Systemic TMA (*n* = 29)	All patients (*N* = 35)	*P*-value^a^
Outcome at 3 mo				
Serum creatinine level, mg/dl	2.4 (1.9–2.4)	1.9 (1.3–3.0)	2.0 (1.4–2.4)	0.18
UPCR, g/g	1.5 (0.3–3.3)	0.8 (0.2–1.3)	0.9 (0.3–2.0)	0.46
Last follow-up				
Follow-up time (mo)	47 (16–55)	21 (5–47)	22 (6–48)	0.25
Serum creatinine level, mg/dl	2.1 (1.9–3.1)	1.6 (1.2–1.9)	1.8 (1.3–2.2)	0.04
UPCR, g/g	0.7 (0.5–7.7)	0.5 (0.2–0.9)	0.5 (0.2–1.0)	0.17
Death	0/6 (0%)	3/29 (10%)	3/35 (9%)	0.99
Graft loss censored for death	0/6 (0%)	9/29 (31%)	9/35 (26%)	0.30
Transplantectomy (refractory TMA)	0/6 (0%)	3/29 (10%)	3/35 (9%)	0.99
Acute rejection	0/6 (0%)	1/29 (3%)	1/35 (3%)	0.99

TMA, thrombotic microangiopathy; mo, month; UPCR, urine protein-to-creatinine ratio.

Categorical variables are described as numbers (%) and continuous variables are described as median (interquartile range), as appropriate.

a *P*-value represents tests of significance from Mann-Whitney test or Fisher exact test, as appropriate.

Based on univariate analysis, dialysis requirement, elevated serum creatinine level at diagnosis of STEC-HUS and systemic TMA were significant risk factors for graft loss (Supplementary Table S4). The use of different treatment strategies did not seem to influence the graft outcome (Supplementary Table S4).

## Discussion

To date, the present study is the largest case series of STEC-HUS in the specific population of adult KTRs. We aimed to determine the clinical, biological, and microbiological features of STEC-HUS at initial presentation and assess the subsequent kidney transplant and recipients’ outcome.

### Underdiagnosis of STEC-HUS in KTRs

Our study first highlights that STEC-HUS is not so rare among KTRs. Indeed, we measured an incidence rate of 14.4 per 100,000 KTR-yrs between 2012 and 2022, as compared with an annual incidence rate of 1 per 100,000 children aged < 15 years in France during a similar period.^21^ Besides, KTRs with STEC-HUS may exhibit unusual clinical presentation, because of the absence of prodromic diarrhea or isolated proteinuria without AKI, rendering its diagnosis more challenging. Patients may present with localized TMA, a milder form than systemic TMA as previously described in the posttransplant setting.^22^^,^^23^ We speculate that some cases of localized TMA may have been undiagnosed in patients presenting with AKI associated with STEC infection who further recovered without graft biopsy. Considering these data, STEC-HUS probably remains underdiagnosed in KTRs. Therefore, we recommend that STEC-HUS should be systematically considered in any case of *de novo* posttransplant TMA as a differential diagnosis,^2^^,^^4^^,^^5^ including in the presence of isolated intrarenal TMA and/or in the absence of prodromic diarrhea.^5^

### Severity of STEC-HUS in KTRs

The importance of diagnosing STEC-HUS particularly relies on its severity in KTRs in terms of both clinical presentation and outcome, a second major point raised by our study. Indeed, we found a large proportion of patients experiencing severe extrarenal manifestations in the acute phase, that is, neurological and cardiac involvement, commonly known as life-threatening complications related to native STEC-HUS in both adults and children.^11^^,^^24^ These complications (neurological and cardiac) do not seem specific to the population of KTRs, because they concerned 76% and 43% of patients, respectively in a recent French cohort study which mainly included immunocompetent adults.^11^ Unexpectedly, STEC-HUS led to graft loss in one-quarter (26%) of patients in a median time of 19 days, which is considerable. As a comparison, 10% of patients remained dialysis-dependent following STEC-HUS episode in the French cohort study.^11^ This difference with our finding may be explained by multiple intricate factors, including an impaired renal function preceding the occurrence of STEC-HUS in KTRs, the immunocompromised status, the increased susceptibility of kidney graft to Stx-mediated endothelial damage, and the association with other causative factors favoring TMA such as CNIs. In the era of eculizumab therapy, STEC-HUS appears as a more deleterious complication than aHUS recurrence in KTRs,^25^ pinpointing the urgent need for diagnosis improvement and effective therapies.

### Predictive Factors for Severity and Outcome of STEC-HUS in KTR

Considering the deleterious impact of STEC-HUS in KTRs, the determination of predisposing factors for disease severity and outcome appears crucial in this population. The configuration of our study did not allow us to address this question that obviously requires us to include matched KTR controls with no STEC-HUS and a larger sample, which remains challenging because of the scarcity of the disease. Regarding initial severity, systemic TMA appeared associated with increased likelihood of extrarenal manifestations and a more severe AKI, echoing previous findings in KTR with *de novo* posttransplant TMA related to CNI toxicity,^22^^,^^23^ acute rejection, and pregnancy.^23^ Regarding the outcome, we identify dialysis requirement, initial serum creatinine level, and systemic TMA as significant predictors of graft loss. These are in line with previous studies showing length of anuria and prolonged dialysis as redundant risk factors for deleterious kidney prognosis in children with STEC-HUS, encompassing development of chronic kidney disease and end-stage renal disease,^26^^,^^27^ as well as the known deleterious impact of systemic TMA in other forms of posttransplant TMA.^22^^,^^23^ Conversely, a recent paper including 92 kidney graft biopsies with *de novo* TMA from various causes did not find significant difference in graft survival according to the 2 TMA forms.^28^ Beyond renal outcome, the need for RRT was found as a significant predictive factor of death following STEC-HUS in the French cohort, in which 90% of patients who died initially required dialysis.^11^

### The Potential Role of Complement in STEC-HUS Occurrence and Severity in KTRs

The specific contribution of complement dysregulation in STEC-HUS occurrence and severity is also a pending question. Previous case reports found complement gene mutations (C3, CD46, and complement factor I) in KTRs presenting with STEC-HUS,^29^^,^^30^ underscoring a genetic predisposition and the importance of genetic screening prior to kidney transplantation. Interestingly here, 17% of patients had undetermined native nephropathy and comprehensive complement exploration was not systematically performed; thus, we could not completely rule out an underlying predisposing role of complement dysregulation. Although complement abnormalities have been evidenced in experimental and human STEC-HUS,^31^, ^32^, ^33^, ^34^ clinical studies examining their impact on disease severity yield discrepant results. In a French study enrolling 108 children with STEC-HUS, the presence of complement pathogenic variants and increased levels of soluble C5b-9 did not significantly influence dialysis requirement; neither did the prevalence of neurological manifestations during the acute phase nor the development of chronic kidney disease during follow-up.^35^ Conversely, decreased C3 serum levels correlated with the need for RRT and the development of severe extrarenal complications in another pediatric cohort.^36^ We also believe that screening for genetic and acquired complement defects merits to be performed in any case of *de novo* posttransplant TMA, in order to differentiate between cases of aHUS triggered by STEC-HUS infection and cases of STEC-HUS infection potentially aggravated by these defects. The recent availability of ultrarapid sequencing techniques, delivering results within a matter of days, enables the early identification of genetic susceptibilities to guide targeted therapeutic interventions.^37^

### Therapeutic Management of STEC-HUS in KTRs

Another crucial point was the highly heterogenous therapeutic management proposed to KTRs with STEC-HUS among the different centers. This is probably because of the absence of a clearly defined and efficient therapeutic strategy of this entity in the general population,^38^ combined with the complexity of managing the risk of acute rejection related to the need for changing the immunosuppressive regimen in some patients. CNIs are known to induce TMA lesions through complex mechanisms of endothelial injury.^39^ A number of case series have shown that successful conversion to belatacept resulted in the improvement of graft survival in CNI-induced TMA^40^, ^41^, ^42^, ^43^ as well as *de novo* or recurrent aHUS.^44^, ^45^, ^46^ Interestingly in our study, belatacept-based therapy was more prevalent in patients with localized TMA at STEC-HUS diagnosis and graft loss only concerned 2 patients among the 12 who were converted to belatacept, although its use was not associated with better graft outcome. Additional larger studies are warranted to assess its beneficial role as a rescue therapy in posttransplant STEC-HUS.

Based on existing data on the pathogenic role of complement in STEC-HUS, eculizumab has been used in isolated pediatric cases of severe STEC-HUS, reporting favorable neurological, cardiac, and renal outcome.^47^, ^48^, ^49^ In ECULISHU, a recent French phase 3 randomized placebo-controlled trial enrolling 100 children with nonsevere STEC-HUS, the early use of eculizumab did not significantly reduce the need for RRT in the acute phase and did not improve renal function at 2 months.^50^ Interestingly though, eculizumab significantly reduced long-term renal sequelae at 1 year, a secondary composite end point including hypertension, decreased estimated glomerular filtration rate < 90 ml/min per 1.73 m^2^ and proteinuria,^50^ a finding difficult to extrapolate to either our distinct population of immunocompromised adults or severe patients, because multiorgan involvement was an exclusion criteria. Of note in our study, 6 graft losses occurred among the 14 patients treated with eculizumab.

The beneficial effects of azithromycin have already been strongly supported by experimental models, relying on the reduction of *in vitro* Stx production and mortality rate in animal models of STEC-HUS.^51^^,^^52^ Despite this, azithromycin was administered only to 16 patients (46%) in our study; this low rate is probably explained by the current controversy regarding prescription of antibiotics in the setting of STEC-HUS, because different classes, primarily quinolones, and cotrimoxazole, have clearly been demonstrated to trigger the release of Stx *in vitro*.^52^^,^^53^ The results of ZITHROSHU study (NCT02336516), a French prospective multicenter placebo-controlled randomized trial including children with STEC-HUS-related AKI, will assess the specific effect of azithromycin on renal outcome (primary end point: estimated glomerular filtration rate measured at 1 month following diagnosis of STEC-HUS), and thus may support the choice toward azithromycin therapy in adult KTRs. Based on these data and in the era of stool multiplex PCR, which substantially improves the early detection of STEC, the indication of systematic treatment with azithromycin should also be discussed with KTRs diagnosed with STEC infection with the aim of preventing further STEC-HUS.

## Conclusion

To the best of our knowledge, our study is the largest case series of STEC-HUS in adult KTRs ever reported, showing that STEC-HUS is a severe and not so rare complication in this population with an annual incidence 14 times higher than observed in children. We recommend systematic screening of STEC-HUS in any case of *de novo* posttransplant TMA, including in the absence of prodromic diarrhea or in patients with localized intrarenal TMA. Systemic TMA was associated with higher rates of graft loss and death. We hope our findings will likely pave the way for larger clinical studies aiming at identifying prognostic factors and a standardized therapeutic strategy in the specific population of KTRs.

## Disclosure

All the authors declared no competing interests.

## Supplementary Material

Supplementary File (PDF) **Figure S1.** Quantification of the absolute difference in serum creatinine level between 3 months after diagnosis of STEC-HUS and baseline (A), between last follow-up and baseline (B) within localized TMA group (orange), systemic TMA group (purple) and the overall population (red). Each dot represents one individual patient. Box plots represent the mean difference ± SD for each group. **Table S1.** General clinical data at diagnosis of STEC-HUS. **Table S2.** Extrarenal manifestations at diagnosis of STEC-HUS. **Table S3.** Hematological parameters at diagnosis of STEC-HUS. **Table S4.** Univariate analysis of risk factors for graft loss following STEC-HUS. STROBE statement.
