# Supplementary material for: Shiga Toxin–Producing Escherichia coli–Associated Hemolytic Uremic Syndrome in Adult Kidney Transplant Recipients
Source: Kidney Int Rep. 2025 Aug 11;10(11):3843–54. doi: 10.1016/j.ekir.2025.08.004 (PMC12639813; doi:10.1016/j.ekir.2025.08.004)
Supplement: Supplementary File (PDF) — Figure S1. Quantification of the absolute difference in serum creatinine level between 3 months after diagnosis of STEC-HUS and baseline (A), between last follow-up and baseline (B) within localized TMA group (orange), systemic TMA group (purple) and the overall population (red). Each dot represents one individual patient. Box plots represent the mean difference ± SD for each group. Table S1. General clinical data at diagnosis of STEC-HUS. Table S2. Extrarenal manifestations at diagnosis of STEC-HUS. Table S3. Hematological parameters at diagnosis of STEC-HUS. Table S4. Univariate analysis of risk factors for graft loss following STEC-HUS. STROBE statement. [file mmc1.pdf]

Shiga toxin-producing Escherichia coli-associated hemolytic uremic syndrome in adult kidney transplant recipients

SUPPLEMENTARY MATERIAL

Supplementary Table S1. General clinical data at diagnosis of STEC-HUS

Supplementary Table S2. Extrarenal manifestations at diagnosis of STEC-HUS

Supplementary Table S3. Hematological parameters at diagnosis of STEC-HUS

Supplementary Table S4. Univariate analysis of risk factors for graft loss following STEC-HUS

Supplementary Figure S1. Quantification of the absolute difference in serum creatinine level between 3 months after diagnosis of STEC-HUS and baseline (**A**), between last follow-up and baseline (**B**) within localized TMA group (orange), systemic TMA group (purple) and the overall population (red). Each dot represents one individual patient. Box plots represent the mean difference ± SD for each group.

Supplementary Table S1. Clinical data at diagnosis of STEC-HUS

Characteristics	Localized TMA (n=6)	Systemic TMA (n=29)	All patients (n=35)	*P* Value[a]
**Age at HUS diagnosis (yr)**	58 ± 16	57 ± 12	57 ± 13	0.96
**Time since transplantation (yr)**	3.9 (2.1-6.1)	3 (1.1-6.6)	3 (1.2-6.2)	0.76
**Diagnostic delay[b] (dy)**	15 (10-25)	10 (6-26)	11 (7-23)	0.28
**Maintenance immunosuppression**				
Corticosteroids	6/6 (100%)	22/29 (76%)	28/35 (80%)	0.31
Tacrolimus	4/6 (67%)	27/29 (93%)	31/35 (89%)	0.13
Ciclosporine	0/6 (0%)	1/29 (3%)	1/35 (3%)	0.99
Belatacept	2/6 (33%)	1/29 (3%)	3/35 (9%)	0.07
Mycophenolic acid	6/6 (100%)	25/29 (86%)	31/35 (89%)	0.99
Azathioprine	0/6 (0%)	2/29 (7%)	2/35 (6%)	0.99

Categorical variables are described as numbers (%) and continuous variables are described as mean±SD or median (interquartile range), as appropriate. TMA, thrombotic microangiopathy; HUS, hemolytic uremic syndrome; yr, year; dy, day.
[a]*P* value represents tests of significance from *t* test, Mann-Whitney test or Fisher's exact test, as appropriate. [b]Diagnostic delay is defined as the time between the occurrence of first symptoms and the microbiological diagnosis of STEC-HUS.

Supplementary Table S2. Extrarenal manifestations at diagnosis of STEC-HUS

Characteristics	Localized TMA (n=6)	Systemic TMA (n=29)	All patients (n=35)	*P* Value[a]
**Diarrhea**	**4/6 (67%)**	**27/29 (93%)**	**31/35 (89%)**	**0.13**
Bloody diarrhea	0/6 (0%)	7/29 (24%)	7/35 (20%)	0.31
Non bloody diarrhea	4/6 (67%)	20/29 (69%)	24/35 (69%)	0.99
**Neurological manifestations**	**0/6 (0%)**	**14/29 (48%)**	**14/35 (40%)**	**0.06**
Focal deficiency	0/6 (0%)	2/29 (7%)	2/35 (6%)	0.99
(Extra)-pyramidal syndrome	0/6 (0%)	1/29 (3%)	2/35 (6%)	0.99
Seizures	0/6 (0%)	4/29 (14%)	4/35 (11%)	0.99
Coma	0/6 (0%)	4/29 (14%)	4/35 (11%)	0.99
Other neurological manifestations[b]	0/6 (0%)	9/29 (31%)	9/35 (26%)	0.30
**Cardiac manifestations**	**0/6 (0%)**	**5/29 (17%)**	**5/35 (14%)**	**0.56**
Acute myocardial infarction	0/6 (0%)	1/29 (3%)	1/35 (3%)	0.99
Pericarditis / Tamponade	0/6 (0%)	1/29 (3%)	1/35 (3%)	0.99
Other cardiac manifestations[c]	0/6 (0%)	4/29 (10%)	4/35 (11%)	0.99
**Fever**	**0/6 (0%)**	**7/29 (24%)**	**7/35 (20%)**	**0.31**

Categorical variables are described as numbers (%). TMA, thrombotic microangiopathy.
[a]*P* value represents tests of significance from Fisher's exact test.
[b]Other neurological manifestations include headache, confusion and psychomotor slowing.
[c]Other cardiac manifestations include left ventricular failure and non-specific chest pain.

Supplementary Table S3. Hematological parameters at diagnosis of STEC-HUS

Characteristics	Localized TMA (n=6)	Systemic TMA (n=29)	All patients (n=35)	*P* Value[a]
**Hemoglobin level (g/dL)**	9.5 (8.6-10.3)	9.3 (8.1-11.0)	9.3 (8.2-11.0)	0.96
**Platelet count (10^9 cells/L)**	185 (120-237)	97 (71-137)	107 (76-166)	0.02
**White blood cell count (10^9 cells/L)**	5.7 (3.3-8.1)	6.2 (3.6-9.6)	6.2 (3.6-8.6)	0.74
**CRP (mg/L)**	2.3 (0.8-5.1)	17.5 (4.2-46.8)	13 (2.3-42)	0.02
**Schistocytes on blood smear**	0/6 (0%)	18/29 (62%)	18/35 (51%)	0.008
**LDH (U/L)**	282 (220-330)	462 (381-694)	428 (321-595)	0.0005
**Haptoglobin (g/l)**	0.6 (0.3-0.9)	0 (0-0.1)	0 (0-0.5)	0.0033

Categorical variables are described as numbers (%) and continuous variables are described as median (interquartile range). TMA, thrombotic microangiopathy; CRP, C-reactive protein; LDH, lactate deshydrogenase.
[a]*P* value represents tests of significance from Mann-Whitney test or Fisher's exact test, as appropriate.

Supplementary Table S4. Univariate analysis of risk factors for graft loss following STEC-HUS

Variables	Graft loss (n=9)	No graft loss (n=26)	*P* Value[a]
**Neurological signs**	1/9 (11%)	13/26 (50%)	0.06
**UPCR at diagnosis**	3.4 (1.8-5.0)	1.9 (1.1-4.6)	0.29
**UPCR ≥ 0.5 g/g at diagnosis**	8/9 (89%)	25/26 (96%)	0.45
**Serum creatinine level at diagnosis**	5.0 (3.3-7.7)	2.5 (2.1-2.8)	0.004
**AKI at diagnosis**	8/9 (89%)	23/26 (82%)	0.99
**Systemic TMA**	9/9 (100%)	0/26 (0%)	< 0.0001
***stx1-/stx2+* profile**	6/9 (67%)	15/26 (58%)	0.71
**Diagnosis delay**	9.0 (3.0-31.5)	11 (7.0-24.3)	0.29
**Belatacept-based treatment at diagnosis**	0/9 (0%)	3/26 (12%)	0.55
**Switch to Belatacept**	2/9 (22%)	10/26 (38%)	0.45
**Azithromycin**	3/9 (33%)	13/26 (50%)	0.46
**Eculizumab**	6/9 (67%)	8/26 (31%)	0.11
**Plasmapheresis**	5/9 (56%)	6/26 (23%)	0.10
**RRT requirement**	7/9 (78%)	4/26 (15%)	0.001

Categorical variables are described as numbers (%) and continuous variables are described as median (interquartile range). UPCR, urine protein creatinine ratio; AKI, acute kidney injury; TMA, thrombotic microangiopathy; *Stx*, shiga toxin; RRT, renal replacement therapy.
[a]*P* value represents tests of significance from Mann-Whitney test or Fisher's exact test, as appropriate.

Supplementary Figure S1. Quantification of the absolute difference in serum creatinine level between 3 months after diagnosis of STEC-HUS and baseline (**A**), between last follow-up and baseline (**B**) within "localized TMA" group (orange), "systemic TMA" group (purple) and the overall population (red). Each dot represents one individual patient. Box plots represent the mean difference ± SD for each group.

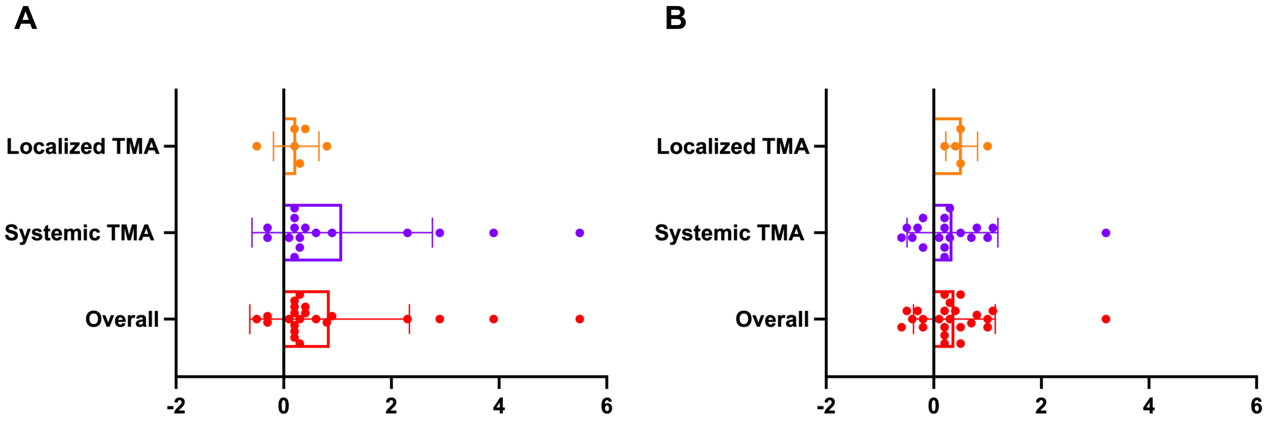

STROBE Statement—checklist of items that should be included in reports of observational studies

	Item No.	Recommendation	Page No.	Relevant text from manuscript
**Title and abstract**	1	(*a*) Indicate the study's design with a commonly used term in the title or the abstract	1	
		(*b*) Provide in the abstract an informative and balanced summary of what was done and what was found	2	
**Introduction**				
Background/rationale	2	Explain the scientific background and rationale for the investigation being reported	3-4	
Objectives	3	State specific objectives, including any prespecified hypotheses	4	
**Methods**				
Study design	4	Present key elements of study design early in the paper	5	
Setting	5	Describe the setting, locations, and relevant dates, including periods of recruitment, exposure, follow-up, and data collection	5-7	
Participants	6	(*a*) *Cohort study*—Give the eligibility criteria, and the sources and methods of selection of participants. Describe methods of follow-up<br>*Case-control study*—Give the eligibility criteria, and the sources and methods of case ascertainment and control selection. Give the rationale for the choice of cases and controls<br>*Cross-sectional study*—Give the eligibility criteria, and the sources and methods of selection of participants		
		(*b*) *Cohort study*—For matched studies, give matching criteria and number of exposed and unexposed<br>*Case-control study*—For matched studies, give matching criteria and the number of controls per case		
Variables	7	Clearly define all outcomes, exposures, predictors, potential confounders, and effect modifiers. Give diagnostic criteria, if applicable	5-7	
Data sources/ measurement	8*	For each variable of interest, give sources of data and details of methods of assessment (measurement). Describe comparability of assessment methods if there is more than one group	5	
Bias	9	Describe any efforts to address potential sources of bias		
Study size	10	Explain how the study size was arrived at		

Continued on next page

Quantitative variables	11	Explain how quantitative variables were handled in the analyses. If applicable, describe which groupings were chosen and why

Statistical methods	12	(*a*) Describe all statistical methods, including those used to control for confounding	7
		(*b*) Describe any methods used to examine subgroups and interactions	
		(*c*) Explain how missing data were addressed	
		(*d*) *Cohort study*—If applicable, explain how loss to follow-up was addressed<br>*Case-control study*—If applicable, explain how matching of cases and controls was addressed<br>*Cross-sectional study*—If applicable, describe analytical methods taking account of sampling strategy	
		(*e*) Describe any sensitivity analyses	
**Results**			
Participants	13*	(a) Report numbers of individuals at each stage of study—eg numbers potentially eligible, examined for eligibility, confirmed eligible, included in the study, completing follow-up, and analysed	8
		(b) Give reasons for non-participation at each stage	
		(c) Consider use of a flow diagram	8
Descriptive data	14*	(a) Give characteristics of study participants (eg demographic, clinical, social) and information on exposures and potential confounders	8-10
		(b) Indicate number of participants with missing data for each variable of interest	
		(c) *Cohort study*—Summarise follow-up time (eg, average and total amount)	
Outcome data	15*	*Cohort study*—Report numbers of outcome events or summary measures over time	10
		Case-control study—Report numbers in each exposure category, or summary measures of exposure	
		Cross-sectional study—Report numbers of outcome events or summary measures	
Main results	16	(*a*) Give unadjusted estimates and, if applicable, confounder-adjusted estimates and their precision (eg, 95% confidence interval). Make clear which confounders were adjusted for and why they were included	10-14 and Tables
		(*b*) Report category boundaries when continuous variables were categorized	
		(*c*) If relevant, consider translating estimates of relative risk into absolute risk for a meaningful time period	

Continued on next page

Other analyses	17	Report other analyses done—eg analyses of subgroups and interactions, and sensitivity analyses	
**Discussion**			
Key results	18	Summarise key results with reference to study objectives	12-17
Limitations	19	Discuss limitations of the study, taking into account sources of potential bias or imprecision. Discuss both direction and magnitude of any potential bias	12-17
Interpretation	20	Give a cautious overall interpretation of results considering objectives, limitations, multiplicity of analyses, results from similar studies, and other relevant evidence	12-17
Generalisability	21	Discuss the generalisability (external validity) of the study results	
**Other information**			
Funding	22	Give the source of funding and the role of the funders for the present study and, if applicable, for the original study on which the present article is based	

*Give information separately for cases and controls in case-control studies and, if applicable, for exposed and unexposed groups in cohort and cross-sectional studies.

Note: An Explanation and Elaboration article discusses each checklist item and gives methodological background and published examples of transparent reporting. The STROBE checklist is best used in conjunction with this article (freely available on the Web sites of PLoS Medicine at http://www.plosmedicine.org/, Annals of Internal Medicine at http://www.annals.org/, and Epidemiology at http://www.epidem.com/). Information on the STROBE Initiative is available at www.strobe-statement.org.
